# Epigenetic silencing of *NTSR1* is associated with lateral and noninvasive growth of colorectal tumors

**DOI:** 10.18632/oncotarget.5034

**Published:** 2015-08-17

**Authors:** Seiko Kamimae, Eiichiro Yamamoto, Masahiro Kai, Takeshi Niinuma, Hiro-o Yamano, Masanori Nojima, Kennjiro Yoshikawa, Tomoaki Kimura, Ryo Takagi, Eiji Harada, Taku Harada, Reo Maruyama, Yasushi Sasaki, Takashi Tokino, Yasuhisa Shinomura, Tamotsu Sugai, Kohzoh Imai, Hiromu Suzuki

**Affiliations:** ^1^ Department of Molecular Biology, Sapporo Medical University School of Medicine, Sapporo, Japan; ^2^ Department of Gastroenterology, Rheumatology, Clinical Immunology, Sapporo Medical University School of Medicine, Sapporo, Japan; ^3^ Department of Gastroenterology, Akita Red Cross Hospital, Akita, Japan; ^4^ Center for Translational Research, The Institute of Medical Science, The University of Tokyo, Tokyo, Japan; ^5^ Medical Genome Science, Research Institute for Frontier Medicine, Sapporo Medical University School of Medicine, Sapporo, Japan; ^6^ Department of Molecular Diagnostic Pathology, Iwate Medical University, Morioka, Japan; ^7^ Center for Medical Innovation, The Institute of Medical Science, The University of Tokyo, Tokyo, Japan

**Keywords:** colorectal tumor, invasion, LST, DNA methylation, biomarker

## Abstract

Our aim was to identify DNA methylation changes associated with the growth pattern and invasiveness of colorectal cancers (CRCs). Comparison of the methylation statuses of large (≥20 mm in diameter along the colonic surface) noninvasive tumors (NTs) and small (<20 mm in diameter along the colonic surface) invasive tumors (ITs) using CpG island microarray analysis showed neurotensin receptor 1 (*NTSR1*) to be hypermethylated in large NTs. Quantitative bisulfite pyrosequencing revealed that *NTSR1* is frequently methylated in colorectal tumors, with large NTs exhibiting the highest methylation levels. The higher *NTSR1* methylation levels were associated with better prognoses. By contrast, *NTSR1* copy number gains were most frequent among small ITs. Methylation of *NTSR1* was associated with the gene's silencing in CRC cell lines, whereas ectopic expression of *NTSR1* promoted proliferation and invasion by CRC cells. Analysis of primary tumors composed of adenomatous and malignant portions revealed that *NTSR1* is frequently methylated in the adenomatous portion, while methylation levels are generally lower in the cancerous portions. These results suggest that *NTSR1* methylation is associated with lateral and noninvasive growth of colorectal tumors, while low levels of methylation may contribute to the malignant potential through activation of *NTSR1*. Our data also indicate that *NTSR1* methylation may be a prognostic biomarker in CRC.

## INTRODUCTION

Colorectal cancer (CRC) is thought to arise through the accumulation of multiple genetic and epigenetic alterations, leading to the activation of oncogenes and loss of function of tumor suppressor genes [1]. Cumulative evidence suggests that numerous changes in DNA methylation act as premalignant steps toward malignancy during colorectal tumorigenesis, and that aberrant CpG island methylation is a major mechanism driving these epigenetic alterations [2, 3]. A subset of CRCs is known to exhibit concurrent hypermethylation of multiple CpG islands, which is referred to as the CpG island methylator phenotype (CIMP) [4]. In addition, a number of studies have shown that aberrant DNA methylation is involved in determining tumor invasiveness or its tendency toward metastasis in CRC [5–8].

Most CRCs are thought to develop through the adenoma-carcinoma sequence, though a subset of CRCs develop from nonpolypoid lesions. Laterally spreading tumors (LSTs) are generally defined as lesions that extend laterally along the luminal wall and are greater than 10 mm in diameter with a low vertical axis [9]. These flat lesions are thought to be less invasive because they are more likely to be found at the adenoma stage or an early CRC stage [9–11]. LSTs are usually categorized into two types: tumors with granular morphology (LST-G) and those with flat or nongranular morphology (LST-NG) [12]. Although the molecular mechanism underlying LST development is not fully understood, there have also been multiple reports of frequent *KRAS* mutation in LSTs [13–15]. And there is reportedly a high prevalence of CIMP-high in this type of tumor [14]. In addition, one recent study reported that LST-G is characterized by an intermediate-methylation epigenotype and *KRAS* mutation, while LST-NG is associated with a low-methylation epigenotype and frequent *CTNNB1* mutation [16]. An inverse association between *APC* methylation and submucosal invasion by LSTs has also been reported [17].

The above-summarized findings indicate epigenetic alterations may be an important determinant of the lateral or vertical growth pattern and invasiveness of colorectal neoplasms. To identify these molecular alterations, we carried out high-throughput CpG island methylation profiling in sets of tumors with lateral or vertical growth. We identified a number of CpG islands differentially methylated between the two groups, including that of *NTSR1*, which was preferentially methylated in laterally growing and noninvasive colorectal tumors. We show that methylation-associated silencing of *NTSR1* is inversely associated with the invasiveness of colorectal tumors, and that its methylation could be a predictive biomarker of a better prognosis in CRC patients.

## RESULTS

### Identification of *NTSR1* methylation in large noninvasive colorectal tumors

**Figure 1:**
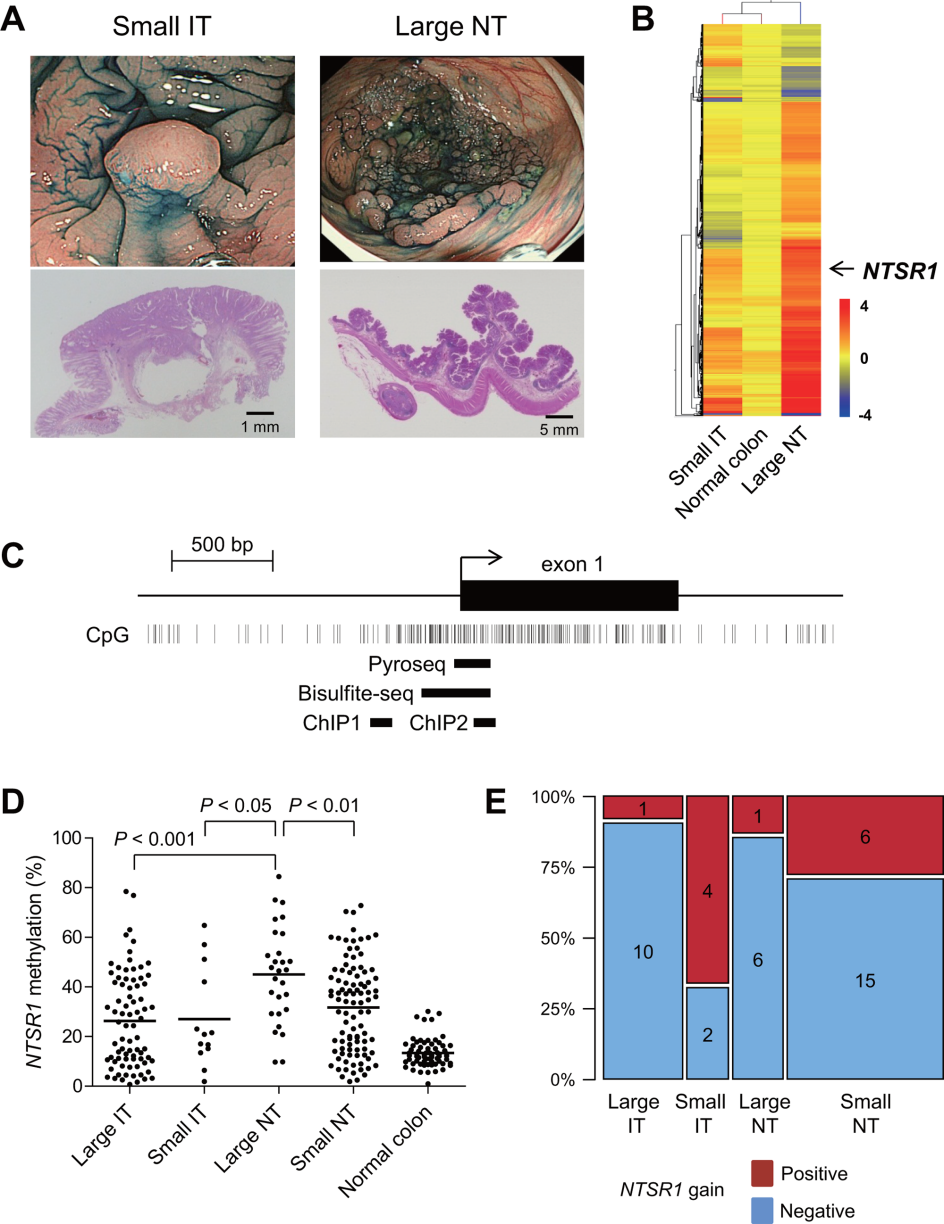
Identification of *NTSR1* methylation in large noninvasive tumors **A.** Representative examples of a small invasive tumor (IT) and a large noninvasive tumors (NT). Endoscopic views are shown on the top and histological views are blow. **B.** Summary of MCAM results from small ITs, large NTs and normal colon. Genes differentially methylated between small ITs and large NTs were selected, after which unsupervised hierarchical clustering was carried out. Each row represents a single probe, and each column represents an average of each category. **C.** Diagram of the *NTSR1* CpG island. The transcription start site and exon 1 are shown on the top, and the regions analyzed using bisulfite pyrosequencing, bisulfite sequencing and ChIP-PCR are shown below. **D.** Summarized results of the bisulfite pyrosequencing analysis of *NTSR1* in specimens from the indicated colorectal tumor types and adjacent normal colonic tissue. **E.** Frequencies of *NTSR1* copy number gain in the indicated types of colorectal tumors. The number of samples in each portion is also shown.

Our first aim was to identify changes in DNA methylation that could be causally related to the growth pattern and invasiveness of colorectal tumors. To accomplish this, we categorized endoscopically or surgically resected colorectal tumors based on the presence or absence of submucosal invasion – i.e., whether they were invasive or noninvasive tumors (ITs or NTs). The tumors were also categorized according to their size, large being ≥ 20 mm in diameter along the colonic surface and small being < 20 mm in diameter along the colonic surface. Then using methylated CpG island amplification-microarray (MCAM) analysis, we assessed the global methylation pattern in large NTs (*n* = 3), small ITs (*n* = 3) and normal colonic mucosa adjacent to the tumors (*n* = 3) (Figure 1A), and screened differentially methylated CpG islands. Unsupervised hierarchical clustering analysis of the MCAM data revealed a substantial number of differentially methylated genes (*n* = 575), the majority of which were methylated at higher levels in large NTs than in small ITs (Figure 1B, Supplementary Table S1). Among these, we focused on neurotensin receptor 1 (*NTSR1*) because although neurotensin (NTS) signaling has been strongly implicated in tumorigenesis, methylation of *NTSR1* has not yet been reported in colorectal tumors (Figure 1B) [18].

We validated the MCAM results by performing quantitative bisulfite pyrosequencing with a set of clinical samples (large IT, *n* = 78; small IT, *n* = 13; large NT, *n* = 28; small NT, *n* = 96; normal colon, n= 66; CRCs in Dukes’ stages C and D were not included) (Figure 1C, 1D). Methylation in the promoter CpG island of *NTSR1* was relatively limited in normal colon but was elevated to differing degrees in primary tumors (Figure 1D). Notably, large NTs showed significantly greater methylation than other tumor types, which is consistent with the MCAM results (Figure 1D). Because expression of *NTSR1* is reportedly upregulated in various malignancies [18], we also determined whether any chromosomal aberrations were associated with *NTSR1* in colorectal tumors. Among the tumor specimens analyzed, array-based comparative genomic hybridization (array CGH) results were available for 45 samples from an earlier study (large IT, *n* = 11; large NT, *n* = 7; small IT, *n* = 6; small NT, *n* = 21) [2]. Those data showed that *NTSR1* copy number gains were substantially more frequent in small ITs than other types of tumors (Figure 1E). These results suggest that elevated levels of *NTSR1* methylation are associated with laterally spreading and noninvasive tumor growth, while lower levels of *NTSR1* methylation are related to smaller tumors with more invasive growth patterns. Our data also suggest that *NTSR1* copy number gain may also be related to the invasive tumor growth.

### Association between *NTSR1* methylation and clinicopathological features

**Figure 2:**
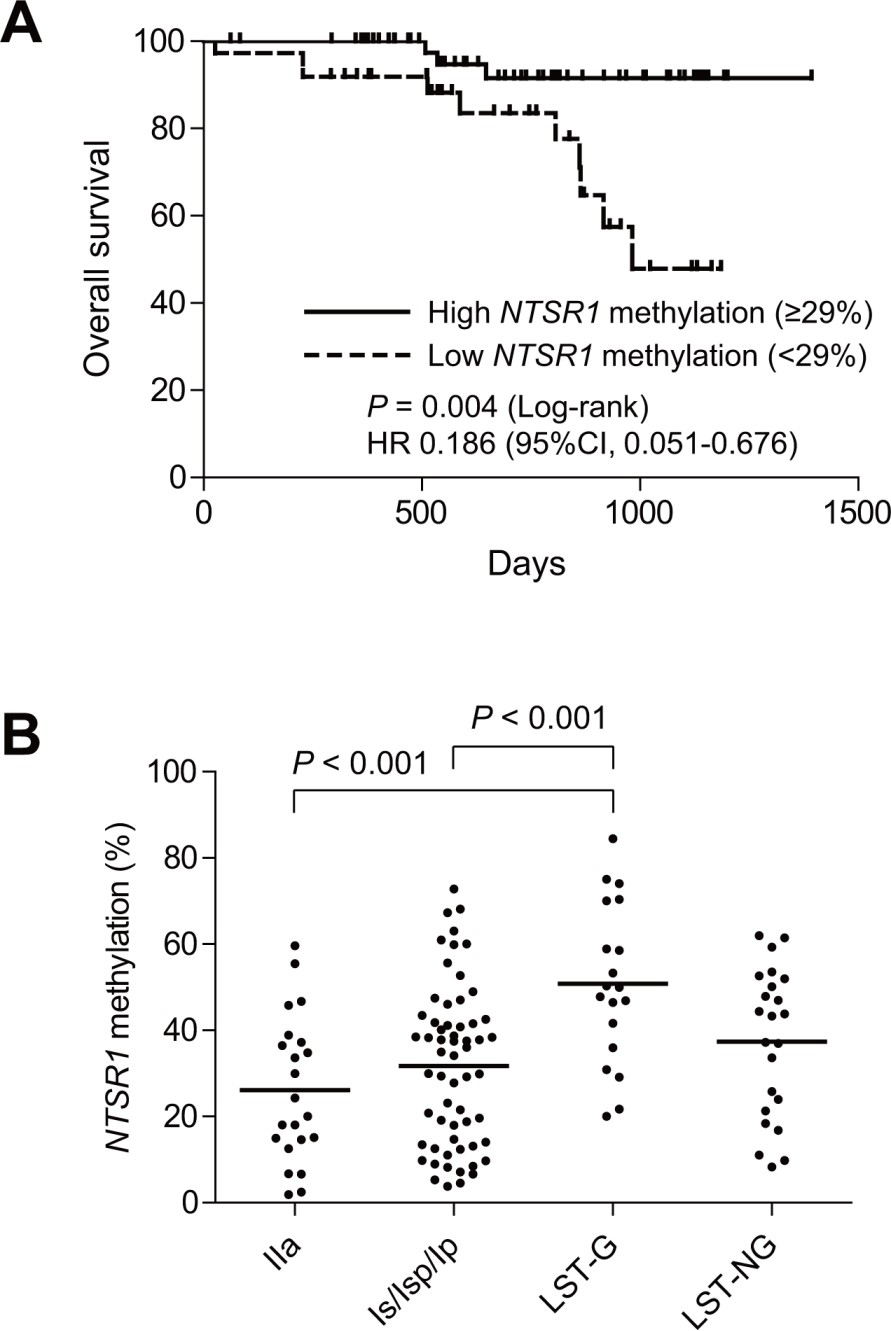
Analysis of *NTSR1* methylation in colorectal tumors **A.** Kaplan-Meier curves showing the effect of *NTSR1* methylation (high, ≥ 29%; low, < 29%) on overall survival of patients with invasive colorectal tumors (*n* = 91). **B.** Summarized results of bisulfite pyrosequencing of *NTSR1* in the indicated morphological types of noninvasive tumors.

We next assessed the association between *NTSR1* methylation and other molecular and clinicopathological features of colorectal tumors. Survival data were available for 91 patients with invasive tumors, and Cox regression analysis revealed the lowest hazard ratio for the high-methylation group (HR = 0.186; 95% CI, 0.051–0.676), when we employed a cutoff value of 29%. Kaplan-Meier analysis showed better overall survival among patients with high *NTSR1* methylation (Figure 2A). We thus categorizing the tumors into high- (≥29%) or low-methylation groups (<29%) based on the level of *NTSR1* methylation, but we detected no statistically significant differences with respect to age, gender or tumor location between the two groups (Tables 1, 2). *NTSR1* methylation also did not correlate with *BRAF* or *TP53* mutation or CIMP status, though *KRAS* mutation was more prevalent in the high-methylation group (Table 1, 2). Among invasive tumors, distant metastasis showed a weak correlation with *NTSR1* methylation, while other clinicopathological characteristics had no significant association (Table 1). Among noninvasive tumors, including precursor lesions and carcinoma in situ, levels of *NTSR1* methylation were significantly higher in LST-G lesions than other morphological types (Figure 2B), but histological type did not correlate with *NTSR1* methylation (Table 2). We further examined the association between *NTSR1* expression and prognosis using a publicly available data set from 62 CRC patients (GSE12945) [19]. When we categorized CRC patients according to their *NTSR1* expression levels, we found shorter overall survival among those with tumors that strongly expressed *NTSR1* (Supplementary Figure S1). These results suggest that higher methylation and weaker expression of *NTSR1* is associated with a better prognosis in CRC patients.

**Table 1:** Correlation between *NTSR1* methylation and the clinicopathological features of invasive colorectal tumors

	*NTSR*1 methylation		*P*
	<29% (*n* = 64)	≥29% (*n* = 67)	
Age (y, mean ± SD)	66.33 ± 12.92	68.81 ± 10.21	NS
Gender			
Female	21	30	NS
Male	43	37	
Location			
Right	29	28	NS
Left	11	23	
Rectum	24	16	
*BRAF*			
Mut	1	5	NS
Wt	63	62	
*KRAS*			
Mut	16	21	NS
Wt	48	46	
*TP53*			
Mut	24	26	NS
Wt	40	41	
CIMP			
Positive	4	10	NS
Negative	60	57	
pT category			
pT1	12	8	NS
pT2	8	13	
pT3	34	41	
pT4	6	5	
NA	4	0	
pN category			
pN0	40	51	NS
pN1	20	15	
NA	4	1	
pM category			
pM0	56	66	0.018
pM1	5	0	
NA	3	1	
Dukes’ stage			
A	18	18	NS
B	22	33	
C	16	15	
D	5	0	
NA	3	1	
Lymphatic invasion			
Negative	5	2	NS
Positive	55	64	
NA	4	1	
Vascular invasion			
Negative	8	6	NS
Positive	51	60	
NA	5	1	

NA, not available; NS, not significant

**Table 2:** Correlation between *NTSR1* methylation and the clinicopathological features of noninvasive colorectal tumors

	*NTSR*1 methylation		*P*
	<29% (*n* = 47)	≥29% (*n* = 77)	
Age (y, mean ±SD)	69.21 ± 9.78	68.91 ± 10.03	NS
Gender			
Female	14	27	NS
Male	33	50	
Location			
Right	24	37	NS
Left	13	16	
Rectum	10	24	
*BRAF*			
Mut	9	8	NS
Wt	38	69	
*KRAS*			
Mut	12	32	NS
Wt	35	45	
*TP53*			
Mut	3	7	NS
Wt	44	70	
CIMP			
Positive	10	15	NS
Negative	37	62	
Pathology			
HP	2	5	NS
TSA	3	9	
SSA	6	5	
T-ad	17	20	
TV-ad	10	22	
Cancer (Tis)	9	16	

NA, not available; NS, not significant

### Methylation of *NTSR1* is associated with gene silencing in CRC cells

**Figure 3:**
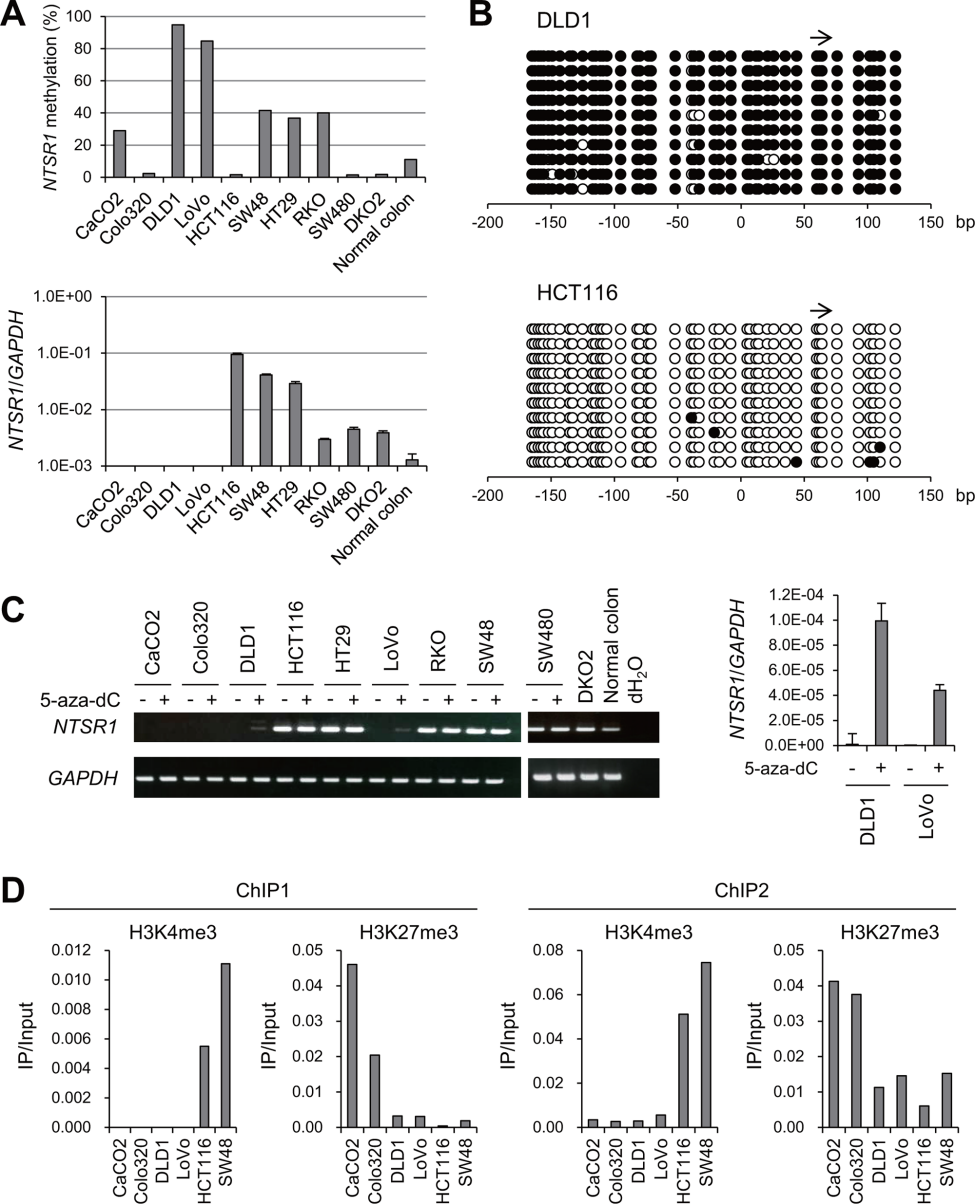
Analysis of *NTSR1* methylation and expression in CRC cell lines **A.** Bisulfite pyrosequencing (top) and quantitative RT-PCR (bottom) analyses of *NTSR1* in the indicated CRC cell lines and normal colonic tissue. **B.** Bisulfite sequencing analysis of the *NTSR1* CpG island in the indicated CRC cell lines. Open and filled circles represent unmethylated and methylated CpG sites, respectively. The arrow indicates the region analyzed with bisulfite pyrosequencing. **C.** RT-PCR results (left) and quantitative RT-PCR (right) analysis of *NTSR1* in the indicated CRC cell lines, with or without 5-aza-dC treatment. Cells were treated with 2 μM 5-aza-dC for 72 h. **D.** ChIP-PCR analyses of *NTSR1* in CRC cells. Levels of H3K4me3 and H3K27me3 in two regions around the *NTSR1* transcription start site (see Figure 1C) are shown.

To determine whether *NTSR1* methylation is associated with the gene's silencing, we analyzed the methylation and expression statuses of *NTSR1* in a series of CRC cell lines (Figure 3A). Various levels of *NTSR1* methylation were detected in 6 of the 9 CRC cell lines tested, with DLD1 and LoVo cells exhibiting the highest levels. Bisulfite sequencing was then used to obtain a more detailed picture of the methylation status in selected cell lines. We found that the CpG island of *NTSR1* is densely methylated in these cells, but is nearly completely unmethylated in HCT116 cells (Figure 3B, Supplementary Figure S2). Reverse transcription-polymerase chain reaction (RT-PCR) analysis revealed a loss of *NTSR1* expression in DLD1 and LoVo cells, which was restored by treatment with the DNA methyltransferase inhibitor 5-aza-2′-deoxycytidine (5-aza-dC), suggesting DNA methylation is responsible for the transcriptional silencing (Figure 3A, 3C, Supplementary Figure S3). By contrast, in 5 of 9 CRC cell lines *NTSR1* expression was higher than in normal colon, irrespective of their methylation levels (Figure 2A). We also analyzed in detail the methylation status of *NTSR1* in SW48 cells, because these cells exhibited a moderate level of DNA methylation and elevated *NTSR1* expression (Figure 3A). Bisulfite sequencing revealed a mixture of methylated and unmethylated alleles in SW48 cells, suggesting the gene is expressed from the unmethylated alleles (Supplementary Figure S2).

*NTSR1* was also not expressed in CaCO2 or Colo320 cells, but we detected low levels of DNA methylation and a lack of re-expression after 5-aza-dC treatment, indicating that a different mechanism may be responsible for the gene silencing (Figure 3A, 3C). We therefore analyzed the histone modification status in selected CRC cell lines. Using chromatin immunoprecipitation (ChIP)-PCR, we assessed trimethylated histone H3 lysine 4 (H3K4me3), an active histone mark, and H3K27me3, a repressive histone mark, in two regions around the *NTSR1* transcription start site (Figure 1C). We found that levels of H3K4me3 were markedly higher in cells in which *NTSR1* was expressed (HCT116 and SW48) than in cells in which *NTSR1* was silenced (Figure 3D). By contrast, H3K27me3 levels were significantly higher in CaCO2 and LoVo cells than other cells, suggesting that a repressive histone modification is involved in the silencing of *NTSR1* in these cells.

To confirm whether DNA methylation is associated with *NTSR1* silencing in primary CRCs, we analyzed data sets from The Cancer Genome Atlas (TCGA). We observed inverse relationships between the methylation levels on multiple probe sets of an Infinium BeadChip and levels of *NTSR1* expression in CRC tissues (Supplementary Figure S4). Taken together, these results suggest that CpG island methylation and histone modifications are associated with the silencing of *NTSR1*.

### Functional analysis of *NTSR1* in CRC cells

**Figure 4:**
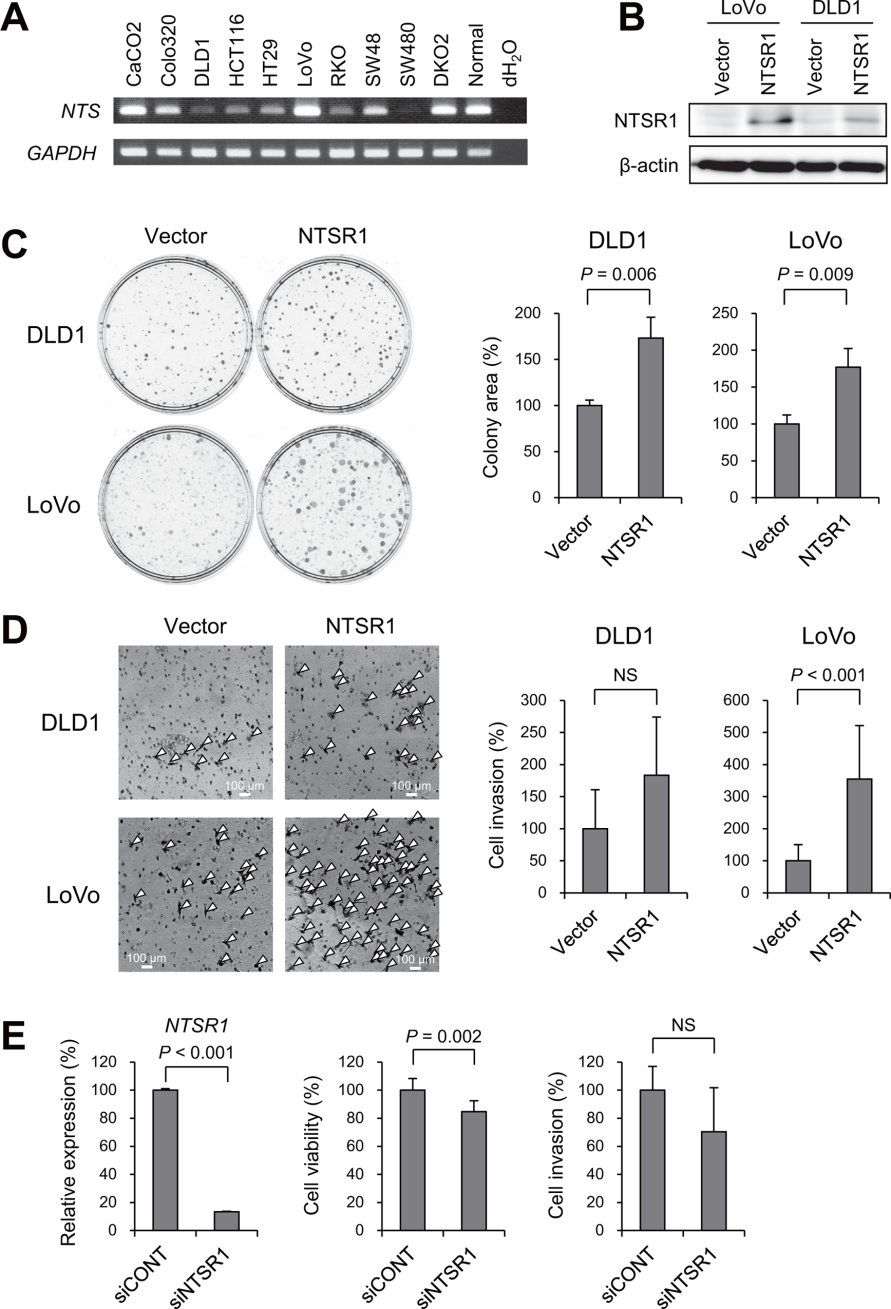
Functional analysis of *NTSR1* in CRC cells **A.** RT-PCR analysis of *NTS* in CRC cell lines and normal colonic tissue. **B.** Western blot analysis of NTSR1 in the indicated CRC cells transfected with a NTSR1 expression vector or a control vector (Vector). **C.** Colony formation assays using the indicated CRC cell lines transfected with a NTSR1 expression vector or a control vector. Representative results are on the left, and relative colony formation efficiencies are on the right. Shown are means of 3 replications; error bars represent SDs. **D.** Matrigel invasion assays using the indicated CRC cell lines transfected with a NTSR1 expression vector or a control vector. Invading cells are indicated by arrowheads. Shown on the right are the means of 5 random microscopic fields per membrane; error bars represent SDs. **E.** Results of quantitative RT-PCR analysis of *NTSR1* expression (left), cell viability assays (middle) and Matrigel invasion assays (right) using HCT116 cells transfected with siRNA targeting *NTSR1* (siNTSR1) or a control siRNA (siCONT). Values were normalized to cells transfected with the control siRNA. Cell viability assay results are means of 8 replications. Matrigel invasion assay results are means of 5 random microscopic fields per membrane. Error bars represent SDs. NS, not significant.

To clarify the function of *NTSR1* in CRC cells, we next assessed the expression of *NTS* in a series of CRC cell lines, most of which exhibited detectable expression in RT-PCR analyses (Figure 4A). We then transfected two cell lines in which *NTSR1* was silenced (DLD1 and LoVo) with a NTSR1 expression vector and performed a flow cytometric analysis. We found that ectopic NTSR1 expression did not induce cell cycle arrest or apoptosis in CRC cells (Figure 4B, Supplementary Figure S5). Colony formation assays showed that cells expressing ectopic NTSR1 formed much larger colonies than those transfected with an empty vector (Figure 4C), and Matrigel invasion assays showed that NTSR1 promoted CRC cell invasion (Figure 4D). Conversely, *NTSR1* knockdown in CRC cells otherwise exhibiting strong *NTSR1* expression (HCT116) suppressed cell proliferation (Figure 4E). Following *NTSR1* depletion, HCT116 cells also showed a tendency toward reduced invasiveness, though the effect was not statistically significant (Figure 4E).

### Alteration of *NTSR1* methylation during the progression of colorectal tumorigenesis

**Figure 5:**
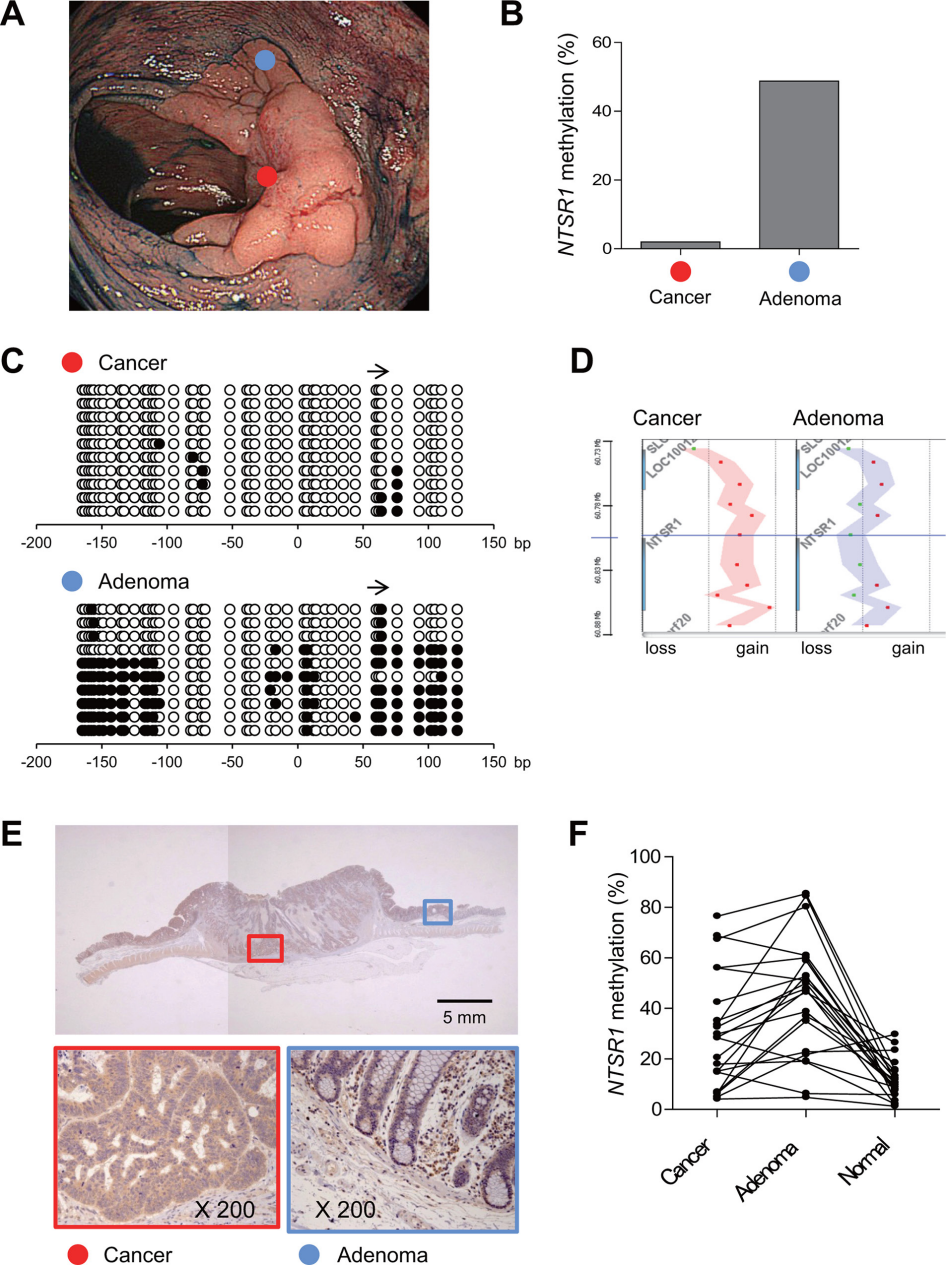
Alteration of *NTSR1* methylation during the progression of colorectal tumorigenesis **A.** Endoscopic view of a colorectal tumor consisting of an adenomatous portion (blue dot) and an invasive cancerous portion (red dot). **B.** Levels of *NTSR1* methylation in biopsy specimens from the indicated portions analyzed by bisulfite pyrosequencing. **C.** Bisulfite sequencing analysis of *NTSR1* in the indicated portions. The arrow indicates the region analyzed with bisulfite pyrosequencing. **D.** Array CGH results of the *NTSR1* locus in the indicated portions. Each dot represents a single probe and genomic gains are indicated by dots on the right; losses are on the left. **E.** Immunohistochemical analysis of NTSR1. Magnified views of the cancerous and adenomatous portions (indicated by red and blue boxes, respectively) are shown below. **F.** Summarized results of bisulfite pyrosequencing of *NTSR1* in colorectal tumors containing adenomatous and cancerous portions (*n* = 22). Methylation levels in adjacent normal colonic tissue from the same patients are also shown.

The results summarized above suggest *NTSR1* is a target of epigenetic silencing in colorectal tumors, despite it functions as an oncogene. This prompted us to examine the genetic and epigenetic alterations occurring to *NTSR1* during tumor progression by analyzing a mixed colorectal lesion consisting of adenomatous and malignant portions (Figure 5A). Using biopsy specimens from adenomatous and cancerous regions, we analyzed *NTSR1* methylation and copy number alterations. Bisulfite pyrosequencing revealed high levels of *NTSR1* methylation in the adenomatous regions and significantly lower levels in the cancerous regions (Figure 5B). This difference in methylation status was further confirmed by bisulfite sequencing (Figure 5C). In addition, array CGH analysis revealed a *NTSR1* copy number gain in only the cancerous component (Figure 5D). Correspondingly, immunohistochemical analysis of the same tumor specimens showed low and elevated levels of NTSR1 protein in the adenomatous and cancerous regions, respectively (Figure 5E, Supplementary Figure S6). Further analysis of *NTSR1* methylation in additional mixed colorectal lesions containing adenomatous and malignant portions (*n* = 22) showed that methylation levels were significantly higher in the adenomas than adjacent normal colonic tissue, but they were generally lower in the malignant portions (Figure 5F). These results suggest that *NTSR1* is a methylation-prone gene in early colorectal lesions, but reduced methylation and may activate *NTSR1* during the malignant progression of colorectal tumorigenesis.

## DISCUSSION

In the present study, we searched for DNA methylation changes associated with the growth pattern of colorectal tumors. We found that the CpG island of *NTSR1* is highly methylated in laterally spreading and noninvasive tumors, that methylation of *NTSR1* is associated with transcriptional silencing of the gene, and that higher levels of the methylation in primary tumors are associated with better patient survival. Functional analysis suggested that *NTSR1* expression promotes tumor growth and invasion, which is consistent with the better prognosis of patients with *NTSR1* methylation.

NTS is a 13-amino acid neuropeptide initially isolated from bovine hypothalamus [20]. Since then it has been determined that NTS is localized mainly in the central nervous system and distal small bowel, and is released from the gut upon ingestion of fats [21, 22]. The physiological functions of NTS include modulation of GI tract motility [23–25], stimulation of intestinal secretion [26, 27] and stimulation of the growth and regeneration of the intestinal epithelial cells [28, 29]. The NTS signal is transmitted via two G protein-coupled receptors (GPCRs), NTSR1 and NTSR2, which respectively exhibit higher and lower affinity for NTS [30].

An abundance of data implicate NTS and NTSR1 in the progression of several cancers, including pancreatic, breast, lung and colorectal cancers [18], and *NTS* is expressed at higher levels in cultured and primary CRC cells than in normal colon [31]. Epithelial crypts isolated from normal human adult colon lack *NTSR1* expression, whereas it is overexpressed in many CRC cell lines [32]. *NTSR1* is a target of the Tcf/β-catenin complex in the Wnt signaling pathway, and activation of *NTSR1* expression is an early event in colorectal tumorigenesis [33]. Another study reported that *NTSR1* expression progressively increases during the process of colorectal tumorigenesis [34]. Overexpression of *NTSR1* is also observed during the progression of inflammatory bowel disease-related oncogenesis [35, 36]. Through NTSR1, NTS signaling mediates cancer cell proliferation, survival, migration and invasiveness by activating a variety of signaling molecules, including protein kinase C (PKC), extracellular signal-regulated kinase 1 and 2 (ERK1/2) and interleukin (IL)-8 [18, 37, 38]. Administration of NTS significantly increases the growth of CRC cell xenografts in mice, and the effect is blocked by SR 48692, a specific NTSR1 antagonist [39]. Moreover, a recent study using *Ntsr1*-deficient mice demonstrated that targeted disruption of *Ntsr1* reduces susceptibility to azoxymethane (AOM)-induced colon tumorigenesis [40].

Given the apparent oncogenic function of *NTSR1*, it seems paradoxical that *NTSR1* would be a target of aberrant DNA methylation in colorectal tumors. Histone modifications also play important roles in epigenetic regulation, and H3K27me3, which is mediated by the polycomb group proteins, is well known to be a repressive mark. Recent studies showed that genes marked by the polycomb complex in embryonic stem (ES) cells have a predisposition toward DNA hypermethylation in cancer [41, 42]. Moreover, many of the genes hypermethylated in adult cancers are held in a transcription-ready state in ES cells by a bivalent chromatin domain containing both active (H3K4me3) and repressive (H3K27me3) histone marks [43]. *NTSR1* is one such gene, as its promoter contains both H3K4me3 and H3K27me3 marks in both mouse and human ES cells [44]. In addition, we observed that H3K27me3 is also involved in the epigenetic silencing of *NTSR1* in CRC cells. Methylation of *NTSR1* has been reported in pancreatic and lung cancers, suggesting *NTSR1* is a methylation-prone gene in tumors of multiple organs [45, 46].

In colorectal lesions that contain both adenoma and carcinoma components, we often observed *NTSR1* hypermethylation in the adenomatous portion and generally lower methylation levels in the cancerous portions. The mechanism underlying the changes of *NTSR1* methylation remains unclear, but what is known suggests several possibilities: (1) precursor cells without *NTSR1* methylation may selectively survive during the malignant progression, (2) methylated CpG island of *NTSR1* in precursor cells may be actively demethylated during the progression from adenoma to carcinoma, and/or (3) the unmethylated *NTSR1* allele may be amplified in cancer cells. Our array CGH results indicate that amplification of the *NTSR1* locus may be one of the mechanisms by which *NTSR1* methylation is reduced in cancer cells. In addition, as mentioned above, *NTSR1* is a target of the Wnt signaling pathway and is transcriptionally activated by the Tcf/β-catenin complex [33]. The progression from early adenoma to invasive carcinoma is associated with an increase of nuclear β-catenin levels [47], which could promote transcriptional activity at *NTSR1* that prevents the *NTSR1* promoter from being methylated in advanced tumors.

In conclusion, we found that *NTSR1* is frequently methylated in colorectal tumors and that elevated levels of *NTSR1* methylation are associated with laterally spreading and noninvasive growth patterns. We also found that levels of *NTSR1* methylation are likely downregulated during the development of CRC, leading to *NTSR1* activation. Because *NTSR1* functions as an oncogene and NTS-NTSR1 signaling is a potential target of anti-cancer therapy, methylation of *NTSR1* could be a predictive biomarker of the responsiveness of CRC patients to such treatment.

## MATERIALS AND METHODS

### Study population and cell lines

Colorectal tumor tissues were collected from Japanese patients who underwent endoscopic or surgical resection at Akita Red Cross Hospital. A total of 309 specimens from 99 precursor lesions, 156 CRCs and 54 samples of adjacent normal tissue were obtained through surgical resection or endoscopic biopsy. Informed consent was obtained from all patients before collection of the specimens. Approval of this study was obtained from Institutional Review Board of Akita Red Cross Hospital and Sapporo Medical University. CRC cell lines (DLD1, LoVo, HCT116, SW48, CaCO2, Colo320, HT29, RKO and SW480) were obtained and cultured as described previously [48]. HCT116 cells harboring genetic disruptions within the *DNMT1* and *DNMT3B* loci (DKO2) are as described [48]. To restore epigenetically silenced genes, cells were treated with 2 μM 5-aza-dC (SIGMA, St Louis, MO, USA) for 72 h, replacing the drug and medium every 24 h. Genomic DNA was extracted using the standard phenol-chloroform procedure. Total RNA was extracted using TRIZOL reagent (Life Technologies, Carlsbad, CA) and then treated with a DNA-free kit (Life Technologies).

### Endoscopic and histological analysis

High-resolution magnifying endoscopes (CF260AZI; Olympus, Tokyo, Japan) were used for all colonoscopic analyses. All detected colorectal tumors were observed at high magnification after staining with indigo carmine dye and 0.05% crystal violet. Surface microstructures were classified according to Kudo's pit pattern classification system [49]. Most often one biopsy specimen was collected from each lesion for the purpose of extracting genomic DNA. However, when at least two components with different surface microstructures were found in a single lesion, biopsy specimens were obtained for each portion, as described previously [2]. The lesions were then treated through endoscopic mucosal resection (EMR), endoscopic submucosal dissection (ESD) or surgical resection, after which histological analyses were carried out. The morphology of noninvasive tumors was determined according to the Paris classification [50]. LSTs were defined as lesions ≥ 10 mm in diameter with a low vertical axis extending laterally along the interior luminal wall [51].

### MCAM analysis

MCAM was performed as described previously [2]. A BioPrime Plus Array CGH Genomic Labeling System (Life Technologies) was used to label MCA amplicons from tumor samples with Alexa Fluor 647, and those from a pooled mixture of normal colonic tissue with Alexa Fluor 555. Labeled MCA amplicons were then hybridized to a custom human CpG island microarray (G4497A; Agilent Technologies, Santa Clara, CA, USA), which included 15,134 probes covering 6,157 unique genes. After washing, the array was scanned using an Agilent DNA Microarray Scanner (Agilent technologies), and the data were processed using Feature Extraction ver. 10.7 (Agilent Technologies) and analyzed using GeneSpring GX ver. 11 (Agilent technologies).

### Methylation analysis using bisulfite-pyrosequencing and bisulfite sequencing

Genomic DNA (1 μg) was modified with sodium bisulfite using an EpiTect Bisulfite Kit (Qiagen, Hilden, Germany), after which bisulfite sequencing and pyrosequencing were carried out as described previously [2]. For bisulfite pyrosequencing, the biotinylated PCR product was purified, made single-stranded and used as a template in a pyrosequencing reaction run according to the manufacturer's instructions. The pyrosequencing reaction was carried out using a PSQ96 system with a PyroGold reagent Kit (Qiagen), and the results were analyzed using Q-CpG software (Qiagen). Tumors were defined as CIMP-positive when methylation was detected in the loci of three or more of five markers: MINT1, MINT2, MINT12, MINT31 and p16 [2]. For bisulfite sequencing, amplified PCR products were cloned into pCR2.1-TOPO vector (Life Technologies), and 10 clones from each sample were sequenced using an ABI3130x automated sequencer (Life Technologies). Primer sequences and PCR product sizes are listed in Supplementary Table S2.

### Mutation analysis

Mutations within codon 600 of *BRAF* and codons 12 and 13 of *KRAS* were examined by pyrosequencing using *BRAF* and *KRAS* pyro kits (Qiagen) according to the manufacturer's instructions [2]. Mutation of *TP53* was initially determined by PCR-SSCP followed by direct sequencing, as described [2].

### RT-PCR

Single-stranded cDNA was prepared using SuperScript III reverse transcriptase (Life Technologies), after which the integrity of the cDNA was confirmed by amplifying glyceraldehydes-3-phosphate dehydrogenase (*GAPDH*). Primer sequences and PCR product sizes are listed in Supplementary Table S2. Quantitative RT-PCR was carried out using TaqMan Gene Expression Assays (*NTSR1*, Hs00901549_m1; *GAPDH*, Hs02758991_g1; Life Technologies) and a 7500 Fast Real-Time PCR System (Life Technologies). SDS ver. 1.4 (Life Technologies) was used for comparative delta Ct analysis.

### ChIP-PCR

ChIP was carried out as described previously [52]. Briefly, 5 × 10^5^ cells were cross-linked with formaldehyde (final concentration is 0.5%) for 10 min at room temperature. After washing, the cells were resuspended in 110 μl of SDS lysis buffer and sonicated. The resultant sheared chromatin was immunoprecipitated overnight at 4°C using 0.2 μg of anti-H3K4me3 (#04–745, Millipore, Billerica, Massachusetts, USA) or 2 μg of anti-H3K27me3 (#9733, Cell Signaling Technology, Danvers, MA, USA) antibody. Before adding the antibodies, 10 μl of the sheared chromatin were saved as input DNA. Samples were incubated for 4 h at 65°C to reverse crosslinking, treated with RNase A and proteinase K, and purified. Input DNA and the immunoprecipitates were then subjected to quantitative PCR analysis using Fast SYBR Green PCR Master Mix (Life Technologies) according to the manufacturer's instructions. Primer sequences and PCR product sizes are listed in Supplementary Table S2.

### Expression vector and siRNA

An expression construct containing full-length NTSR1 (pReceiver-M11-NTSR1) was purchased from Gene Copoeia (Rockville, MD, USA). CRC cells (5 × 10^5^ cells/well in 6-well plates) were transfected with 2.5 μg of the expression vector or empty pcDNA3.1 using Lipofectamine 3000 (Life Technologies) according to the manufacturer's instructions. For RNA interference (RNAi)-mediated knockdown of *NTSR1*, 5 × 10^5^ cells were transfected with 25 pmol of Stealth RNAi siRNA (Life Technologies) or a negative control (Life Technologies) using Lipofectamine RNAiMAX (Life Technologies) according to the manufacturer's instructions.

### Western blot analysis

Samples of total cell lysate (20 μg of protein) were separated using SDS-PAGE (12.5% acrylamide) and transferred to polyvinylidene difluoride membranes (Bio-Rad Laboratories, Hercules, CA, USA). The membranes were then blocked with Block Ace (Dainippon Pharmaceutical, Tokyo, Japan) and incubated with goat anti-NTSR1 polyclonal Ab (1:1000 dilution, C-20; Santa Cruz Biotechnology) and mouse anti-β-actin mAb (1:10000 dilution, clone AC-15; Sigma-Aldrich, St. Louis, MO, USA). The immunoreactive bands were visualized using horseradish peroxidase-conjugated anti-mouse or anti-goat IgG (1:10000 dilution; Jackson ImmunoResearch Laboratories, West Grove, PA, USA) and ECL (GE Healthcare Bio-Sciences KK, Tokyo, Japan). Images were acquired using a chemiluminescent CCD imager (Image Quant LAS 4000; GE Healthcare Bio-Sciences KK, Tokyo, Japan).

### Colony formation assay

Cells were transfected with a NTSR1 expression vector or a control vector as described above. After incubation for 24 h, the transfectants were plated on 60-mm culture dishes and selected for 10 to 14 days in 1.0 mg/ml G418, after which colonies were stained with Giemsa.

### Cell viability assay

Cells were transfected with siRNA as described above. After incubation for 24 h, the transfectants were seeded into 96-well plates to a density of 5 × 10^3^ cells per well and incubated for an additional 72 h. Cell viability assays were then carried out using a Cell Counting kit-8 (Dojindo, Tokyo, Japan) according to the manufacturer's instructions.

### Matrigel invasion assay

Cells were transfected with the expression construct or siRNA as described above. After incubation for 24 h, 1 × 10^5^ transfectants suspended in 500 μl of serum-free culture medium were added to the tops of BD BioCoat Matrigel Invasion Chambers (BD Biosciences, Franklin Lakes, NJ, USA) prehydrated with phosphate buffered saline (PBS), and 700 μl of medium supplemented with 10% fetal bovine serum (FBS) were added to the lower wells of the chambers. After incubation for 24 h, the invading cells were stained with 1% toluidine and counted in 5 randomly selected microscopic fields per membrane.

### Flow cytometry

Cells were transfected with the expression construct as described above. After incubation for 72 h, apoptosis was measured using an ApoScreen Annexin V Apoptosis Kit (Southern Biotech, Birmingham, AL, USA). Briefly, 1 × 10^6^ cells were washed twice in cold PBS and then resuspended in cold binding buffer, after which 10 μL of annexin V-FITC was added to 100 μL of the cell suspension. The mixture was then incubated for 15 min on ice in the dark before addition of 380 μL of cold binding buffer and 10 μL of propidium iodide. For cell cycle analysis, cells were fixed in ethanol, and DNA was stained with propidium iodide. For each sample, data were acquired from a minimum of 1 × 10^5^ cells using a BD FACSCant II (BD Biosciences) with BD FACSDiva software (BD Biosciences), and were analyzed using FlowJo ver. 10 (FlowJo, LLC, Ashland, OR, USA).

### Immunohistochemistry

Specimens were fixed in buffered formalin and embedded in paraffin using routine procedures. Sections (4-μm thick) were prepared, dried, deparaffinized and rehydrated before microwave treatment (H2500, Microwave Processor, Bio Rad) in citrate buffer (pH 6.0) for 5 min. A goat anti-NTSR1 polyclonal Ab (1:100 dilution, C-20; Santa Cruz Biotechnology, Santa Cruz, CA, USA) [53] and an automatic staining machine (Envision(00000FE) system; DAKO, Glostrup, Denmark) were used for immunohistochemical labeling. The slides were counterstained with hematoxylin, then dehydrated and mounted.

### Statistical analysis

To compare differences in continuous variables between groups, *t* tests or ANOVA with post hoc Tukey's tests were performed. Fisher's exact test or chi-squared test was used for analysis of categorical data. Values of *P* < 0.05 (two-sided) were considered statistically significant. To assess the association between survival and methylation levels, Cox regression analyses were performed. Kaplan-Meier curves were plotted to compare survival in two groups stratified based on gene methylation or expression status. The minimum *P*-value method was used to determine the best cutoff value of the *NTSR1* methylation level for survival analysis. After excluding data within the highest and the lowest 10 percentiles, all cutoff values were tested using the log-rank test, and a cutoff value with the lowest *P*-value was determined. Statistical analyses were carried out using JMP ver. 10 (SAS Institute Inc., Cary, NC, USA) and GraphPad Prism ver. 5.0.2 (GraphPad Software, La Jolla, CA, USA).

## SUPPLEMENTARY FIGURES AND TABLES




