# Supplementary material for: Epigenetic silencing of NTSR1 is associated with lateral and noninvasive growth of colorectal tumors
Source: Oncotarget. 2015 Aug 17;6(30):29975–90. doi: 10.18632/oncotarget.5034 (PMC4745776; doi:10.18632/oncotarget.5034)
Supplement: Supplementary file 1 [file oncotarget-06-29975-s001.pdf]

SUPPLEMENTARY FIGURES AND TABLES

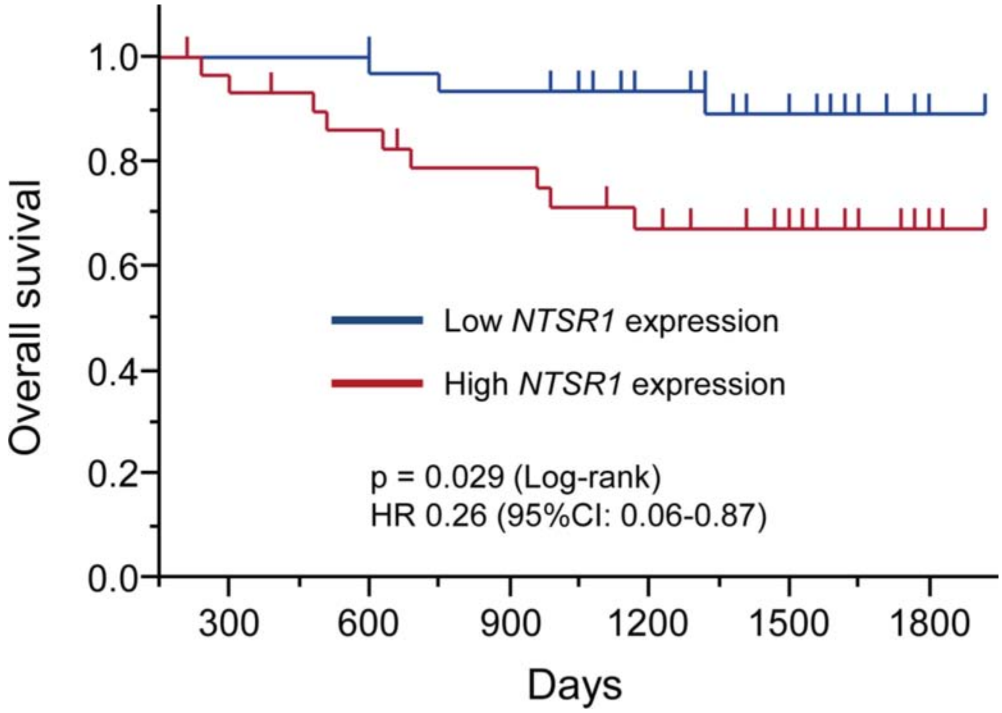

Supplementary Figure S1: Kaplan-Meier curves showing the effect of *NTSR1* expression on overall survival among patients with colorectal tumors.

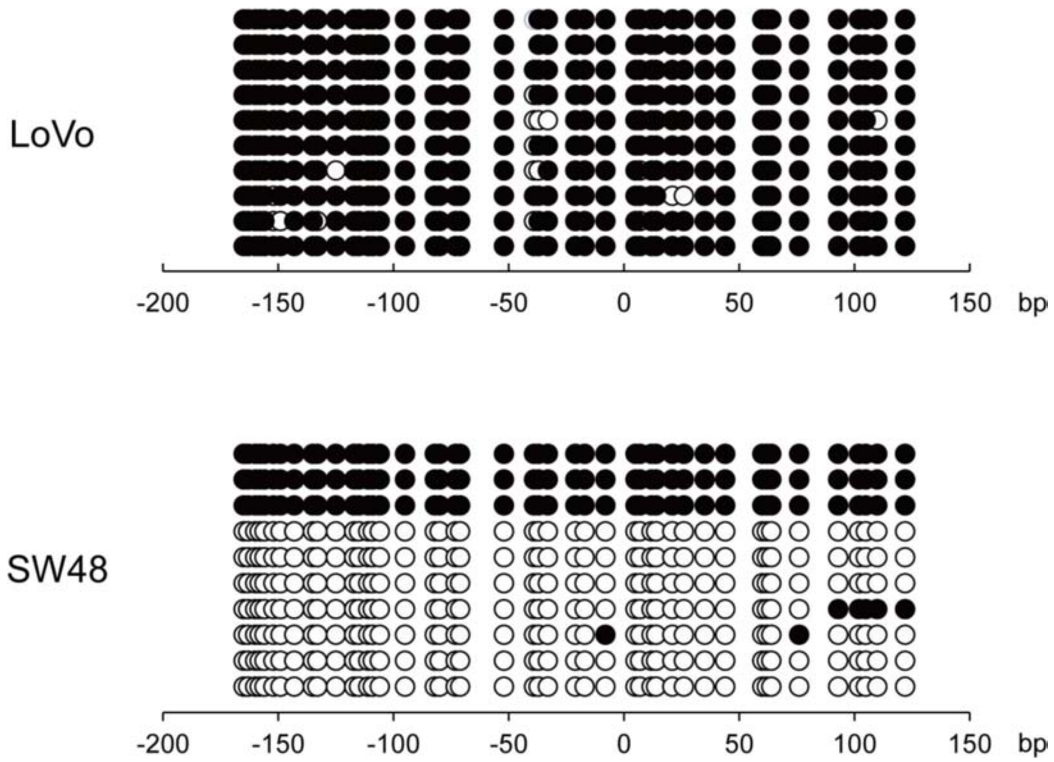

Supplementary Figure S2: Bisulfite sequencing analysis of the *NTSR1* CpG islands in the indicated CRC cell lines. Open and filled circles represent unmethylated and methylated CpG sites, respectively.

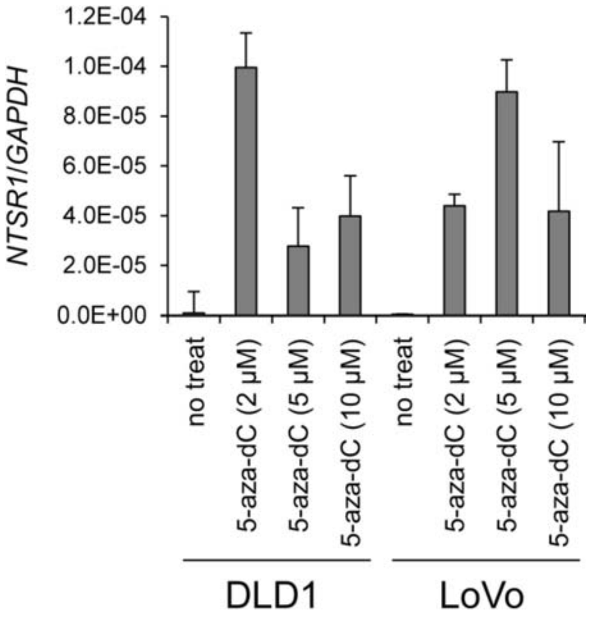

Supplementary Figure S3: Quantitative RT-PCR analysis of *NTSR1* in the indicated CRC cell lines, with or without 5-aza-dC treatment. Cells were treated for 72 h with the indicated concentrations of 5-aza-dC.

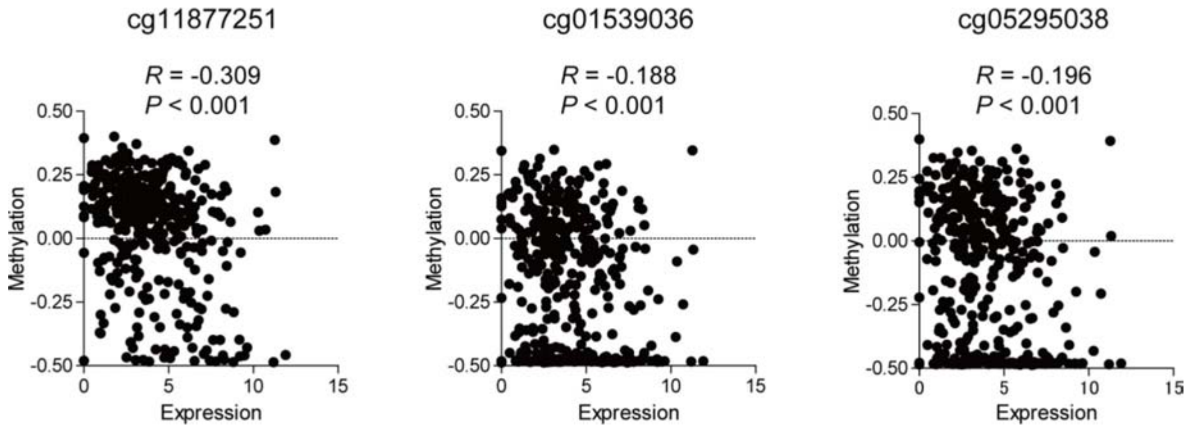

Supplementary Figure S4: Inverse association between DNA methylation and expression of *NTSR1* in primary CRC. Infinium HumanMethylation450 BeadChip data and RNA-seq data were obtained from the Cancer Genomics Browser (https://genome-cancer.ucsc.edu). Three Infinium probe sets within the *NTSR1* CpG island were selected, after which Pearson correlation coefficients were calculated for the relation between β-values and expression levels.

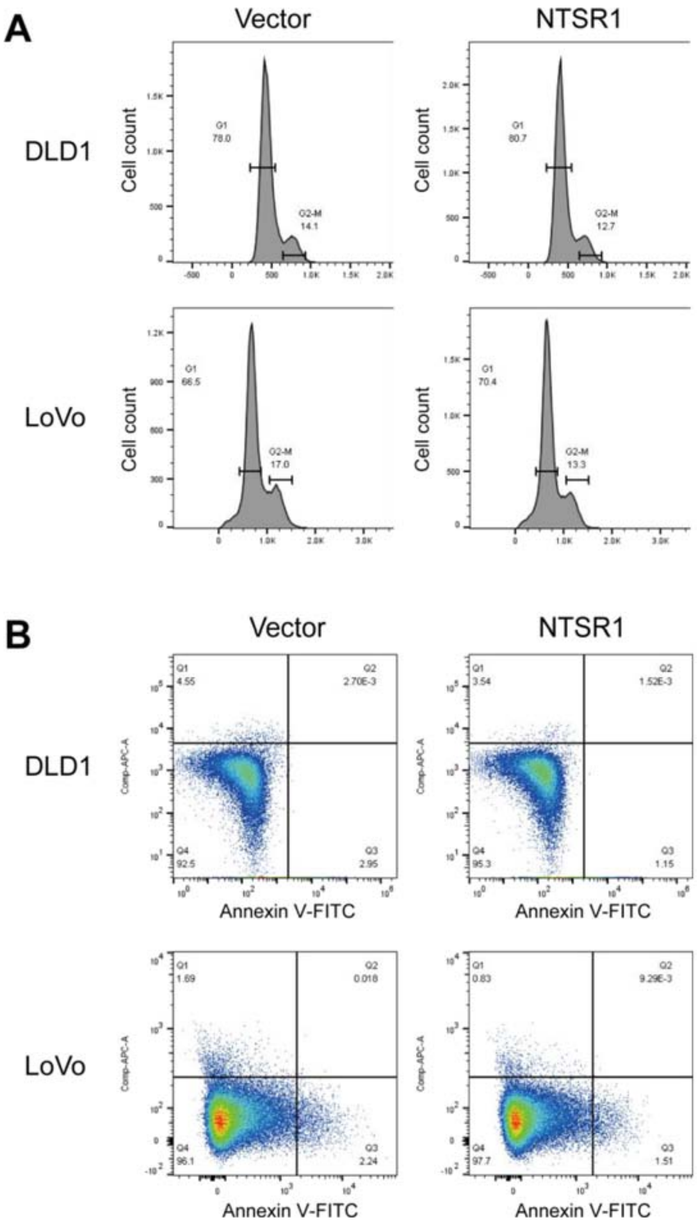

Supplementary Figure S5: A. Representative results of a cell cycle analysis using the indicated cell lines transfected with a NTSR1 expression vector or a control vector. B. Representative results of apoptosis analysis using the indicated cell lines transfected with a NTSR1 expression vector or a control vector.

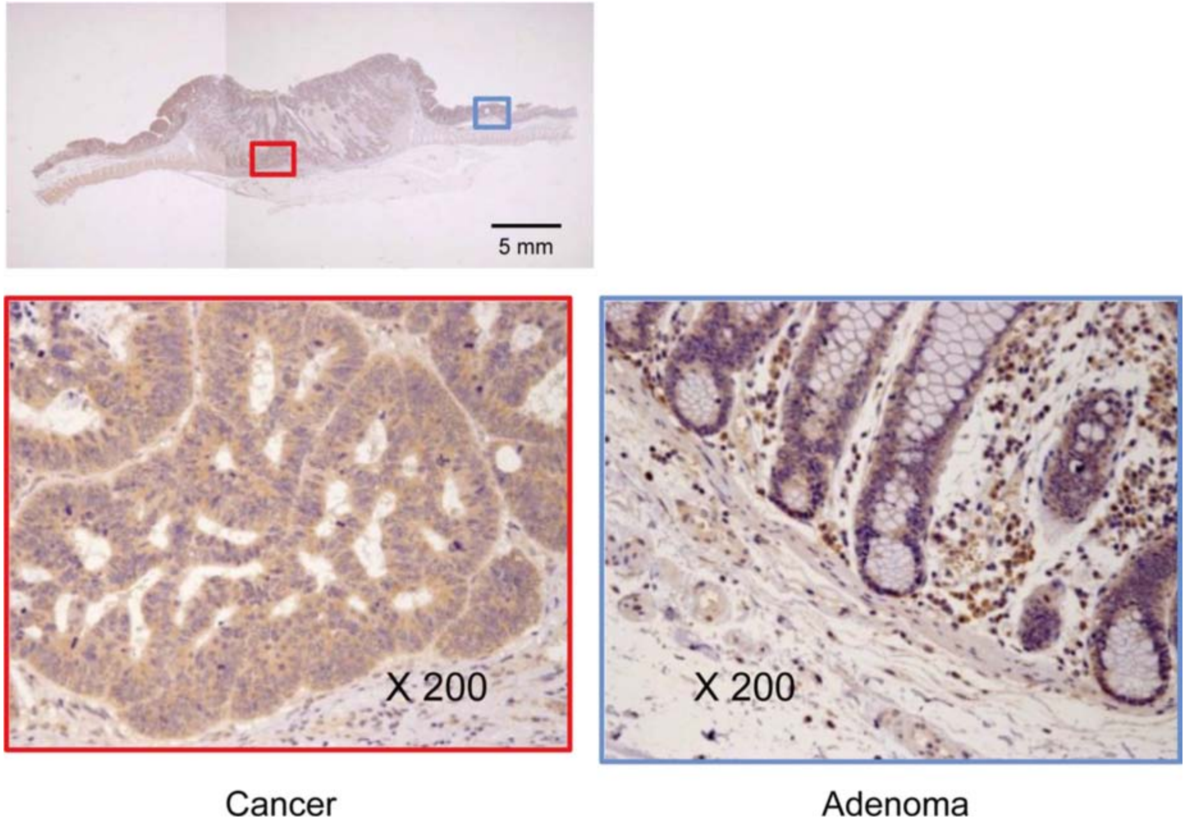

Supplementary Figure S6: Higher magnification images of the immunohistochemical analysis of NTSR1 shown in Figure 5E. Magnified views of the cancerous and adenomatous portions (indicated by red and blue boxes, respectively) are shown below.

Supplementary Table S1: Genes differentially methylated between small ITs and large NTs (determined by MCAM analysis).

Supplementary Table S2: Sequences of the primers used in this study

Primer name	Forward	Reverse	Product size
NTSR1 bisulfite pyroseq	5′-GGGTAGTAGTGYGTGYGTTTTTTT-3′	5′-Bio-CTAACTCCAAAACCCRTAATTCCTC-3′	179 bp
sequencing primer	5′-GGGGGTTTGGGGAAT-3′		
sequence to analyze	YGYGYG		
NTSR1 bisulfite seq	5′-TTTTTTTYGTYGTTATTTTTTATTGTTGGG-3′	5′-CCTAACTCCAAAACCCRTAATTCCTC-3′	343 bp
NTSR1 RT-PCR	5′-GAAGCCGCACCAAGAAGTTCATCA-3′	5′-TCAGCTTGTTGGCGATGATGGTGT-3′	246 bp
NTS RT-PCR	5′-AACAGGAGAAGTTCATGAAGAGGA-3′	5′-CAGTAATAGTAAGAATCTCTTTTGAG-3′	291 bp
NTSR1 ChIP1	5′-TACACCCTCACCCCAACTGA-3′	5′-TCTTCCCTGTTCTCCGCAAT-3′	93 bp
NTSR1 ChIP2	5′-TTTGGAGATCGGAGGCACC-3′	5′-TCCCAGCTTCCGGCTCCTA-3′	97 bp

Y=C or T, R=A or G
